# Urticarial rash as the initial presentation of COVID‐19 infection: A case report

**DOI:** 10.1002/ccr3.6076

**Published:** 2022-07-14

**Authors:** Usamah Al‐Anbagi, Shybin Usman, Abdulqadir J. Nashwan

**Affiliations:** ^1^ Medicine Department, Hazm Mebaireek General Hospital Hamad Medical Corporation Doha Qatar; ^2^ Nursing Department, Hazm Mebaireek General Hospital Hamad Medical Corporation Doha Qatar

**Keywords:** COVID‐19, dermatology, SARS‐CoV‐2, skin, urticarial rashes

## Abstract

During the COVID‐19 pandemic, various skin manifestations have been described. These include an urticarial rash, morbilliform rash, maculopapular rash, vascular lesions, and varicella‐like eruptions. A 30‐year‐old woman presented with a mild cough, then hives and pruritic rash for 3 days, followed by fever, dyspepsia, and throat pain for one day.

## Graphical abstract

The occurrence of cutaneous signs in COVID‐19 is associated with disease severity, although there is inadequate evidence to assess skin manifestations' prognostic relevance. Skin manifestations are often self‐limiting and last six to eight days, although they may persist up to 10 days

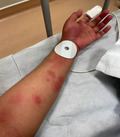

## 1 INTRODUCTION

The *SARS‐CoV‐2* (COVID‐19) pandemic impacted more than 518 million people and killed around 6.3 million patients.^1^ Although the virus is largely responsible for respiratory symptoms, a growing number of dermatological signs and symptoms of this illness have been identified. Several reports worldwide have identified a range of potential skin manifestations of *SARS‐CoV‐2* (COVID‐19).^2^, ^3^, ^4^, ^5^, ^6^, ^7^, ^8^ However, the frequency of dermatological presentation (between 0.2% and 29%) and also the timing of skin manifestations are difficult to ascertain.^9^, ^10^, ^11^

Here, we have a case report of a young woman who initially presented with a generalized pruritic urticarial rash ranging from one to seven centimeters in diameter on January 6, 2022.

## 2 CASE PRESENTATION

A 30‐year‐old woman with no comorbidities and no significant past medical history. She received her third booster dose of mRNA‐1273 COVID‐19 vaccine 5 days before presenting with symptoms.

The initial symptom was a pruritic, erythematous macular rash on the back of her neck and the volar aspect of her forearms. She did not self‐medicate with over‐the‐counter (OTC) medications and sought medical attention on an outpatient basis. She gave a history of mild dry cough one day before developing the rash. On evaluation, she was found to be COVID‐19 Antigen positive and was prescribed antihistamines.

**FIGURE 1:**
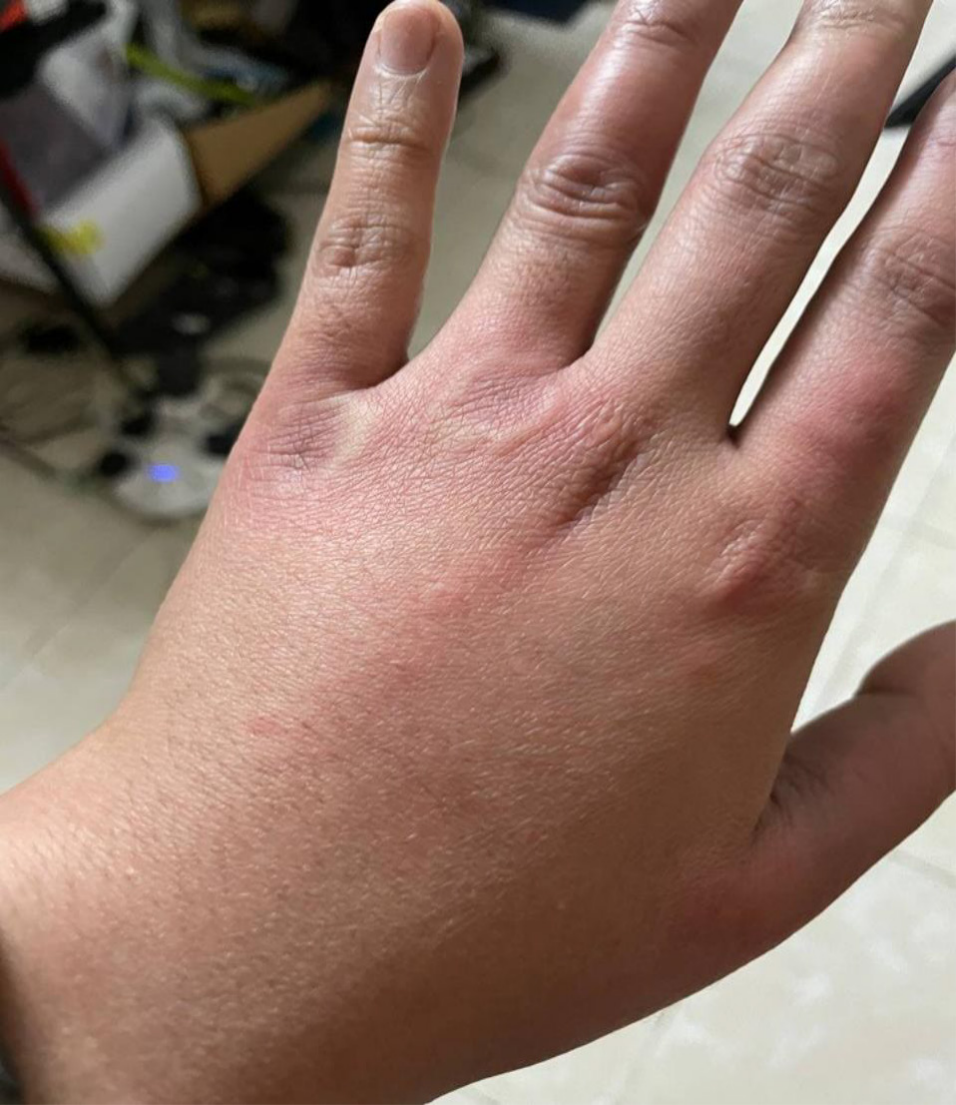
Urticarial rash on the left hand

**FIGURE 2:**
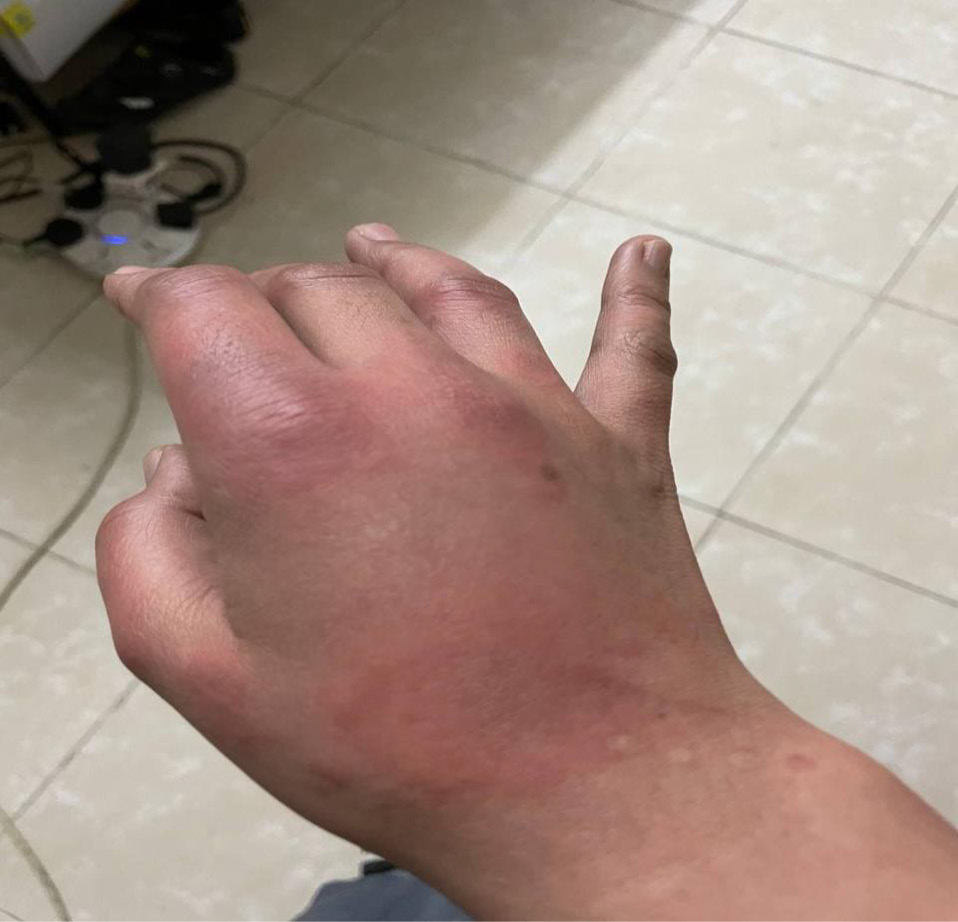
Urticarial rash on the right hand

**FIGURE 3:**
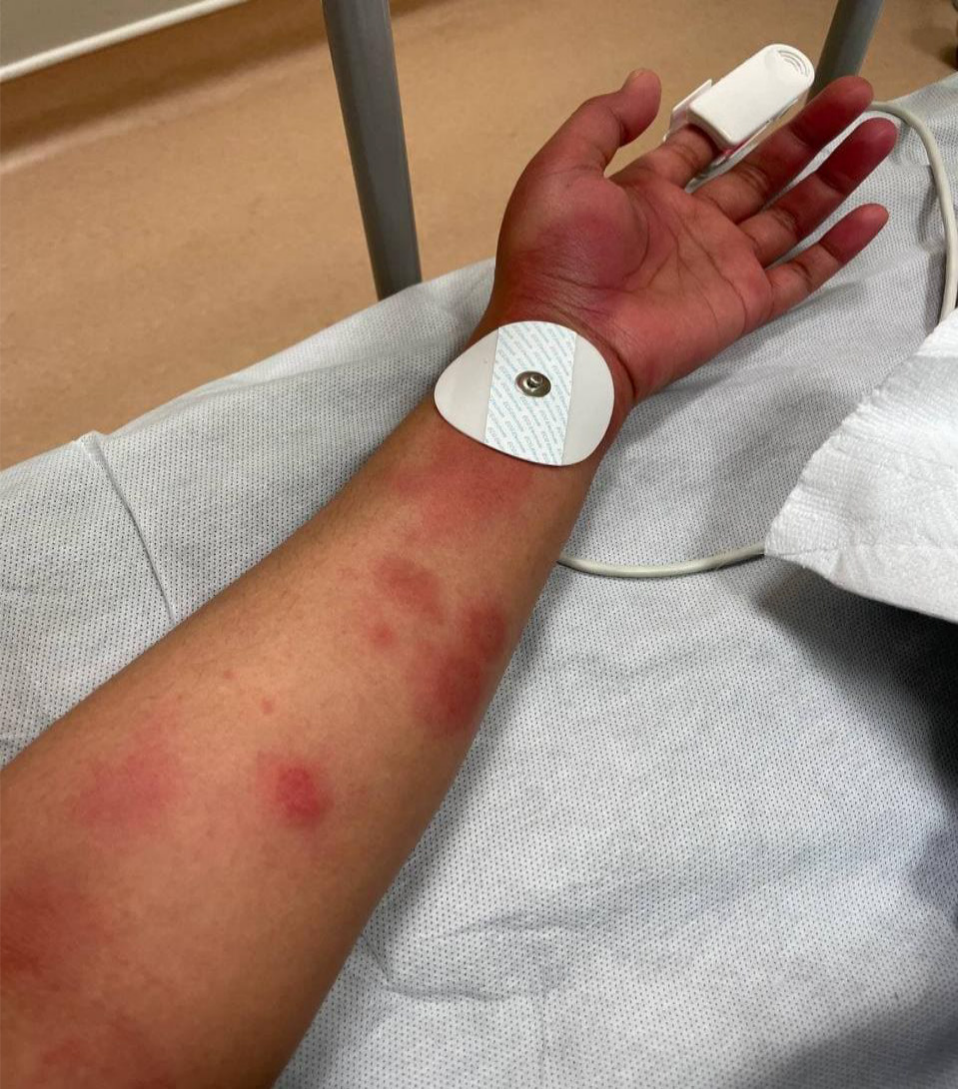
Diffuse urticarial rash on the left upper extremity

On the fourth day of symptoms, she presented to the ED (emergency department) with a worsening pruritic rash, which had spread to involve her neck, face, arms, trunk, and legs (Figures 1, 2, 3) and mild fever, and dyspepsia.

She did not have shortness of breath, stridor, wheeze, chest pain, or abdominal pain. She had no history of insect bites, exposure to new foods, recent use of any medications, over‐the‐counter medications, herbal supplements, and no known disease exposures. The patient denied any past history of similar rashes or allergic reactions and also denied any family history of the similar condition or skin diseases running in her family.

### 2.1 Clinical examination

On examination, the following were the vital signs: on room air, the temp 37.9°C, the blood pressure (BP) 114/72 mmHg, the respiratory rate 20 b/m, the heart rate 85 b/m, and the O_2_ Sat was 99 percent.

She had widespread raised erythematous and pruritic lesions on her face, trunk, and bilateral upper and lower extremities consistent with an urticarial rash (Figures 1, 2, 3). In addition, she had substantial pain as a result of pruritus and a burning‐like feeling in the lesions. The remaining clinical examination, including the respiratory system, was also unremarkable.

COVID‐19 PCR test was positive, Chest X‐ray was unremarkable, and EKG revealed sinus tachycardia only. For the other laboratory investigations (see Table 1).

**TABLE 1:** Laboratory investigations

Laboratory tests	Results	Normal values
White cell count	12 × 10^9^ cells/L	4–10 × 10^9^ cells/L
Red cell count	5.2 × 10^6^/μl	3.8–4.8 × 10^6^/μl
Platelet	367 × 10^3^/μl	150–400 × 10^3^/μl
C reactive protein	129 mg/L	<8.0 mg/L
Serum creatinine	61 μmol/L	44–80 μmol/L
Serum ferritin	308.0 μg/L	12–160 μg/L
Urea	3.0 mmol/L	2.5–7.8 mmol/L
Sodium	137	135–145 mmol/L
Potassium	3.7	3.5–5.3 mmol/L
Bilirubin	27 μmol/L	0–21 μmol/L
Alanine aminotransferase	63 U/L	0–33 U/L
Aspartate aminotransferase	71 U/L	0–32 U/L
Procalcitonin	0.15 ng/ml	<0.5 ng/ml
Interleukin‐6	74 pg/ml	<7 pg/ml

### 2.2 Differential diagnosis

Several etiologies are considered, such as COVID vaccine‐induced, mast cell defects, food‐related allergies, autoimmune conditions, and infectious causes. Because the urticarial rash appeared shortly before her positive PCR test and she had accompanying moderate viral symptoms, the urticaria is thought to be a presenting sign of her COVID‐19 infection. A detailed history was taken and did not reveal any concern for new exposure to food, use of over‐the‐counter medications, recent use of any kind of medications or herbal supplements, as well as no history of a similar attack; and it was unlikely to be an anaphylactic reaction as it was progressive worsening over 3 days duration, other suspected infection were excluded after screening and cultures.

### 2.3 Management and follow‐up

As infective causes were being considered, empirical antibiotic coverage with ceftriaxone 2 g and azithromycin 500 mg once daily were started on by the emergency team during admission. It was discontinued on the third day when preliminary blood culture reports came back negative. Steroids (hydrocortisone IV one dose 200‐mg and then transitioned to oral prednisolone 20 mg, which was given for 4 days) and antihistamines (diphenhydramine 25‐mg injection on need along with oral fexofenadine 180 mg, which was given daily for 5 days then it was replaced with levocetirizine 5‐mg daily dose) and emollient creams were given throughout the hospital stay for the allergic symptoms. Deep venous thrombosis prophylaxis was provided with enoxaparin 40‐mg subcutaneous injection daily. Oral esomeprazole 20‐mg was given for stress ulcer prophylaxis. She was discharged on levocetirizine 5‐mg and esomeprazole 20‐mg once daily for 5 days. She had an apparent improvement of rash after 2 days, with a remaining mild itching and burning sensation. She was discharged home safely on day 6 of hospitalization after all her workup was done, which ruled out other suspected causes of her rashes.

## 3 DISCUSSION

Coronavirus disease (COVID‐19) is caused by the severe acute respiratory syndrome coronavirus 2 virus; which is a ribonucleic acid (RNA) virus that invades host cells through one receptor called angiotensin‐converting enzyme 2 (ACE2) receptor,^12^, ^13^ which is found on small intestine enterocytes, epithelial cells of the lung alveolar, heart, also the endocrine, and neurologic systems.^10^ Multiple cutaneous symptoms have been seen and documented in people infected with COVID‐19. Furthermore, the presence of skin symptoms in COVID‐19 has been observed to vary between 0.2 and 29 percent.^11^ These include an urticarial rash, morbilliform rash, maculopapular rash, livedo reticularis/racemosa‐like pattern, vascular lesions, and varicella‐like eruptions.

The pathogenesis of COVID‐19 urticaria is largely underdiagnosed. Still, it might be mediated by the acute systemic inflammatory response to the COVID‐19 to acute infection, which results in mast cell activation and the production of proinflammatory cytokines.^14^

Skin manifestations associated with COVID‐19 are often self‐limiting and do not lead to complications, and usually, the urticarial rash lasts around 6–8 days and up to 10 days.^2^, ^15^ Urticarial rashes are mostly noted to occur simultaneously or after the onset of noncutaneous symptoms. The presence of skin and dermatological symptoms in COVID‐19 has been linked to disease severity; a Spanish research linked the characteristics of maculopapular or urticarial lesions to the severity of COVID‐19 infection^2^; on the contrary, in their literature study of 21 patients, Sachdeva et al. claimed that there is an implausible link between cutaneous lesions and COVID‐19 infection severity.^15^

Most of the patients achieved adequate symptom control with the use of antihistamines treatment only, which can be used until they get the resolution.^14^ Regarding glucocorticoids use, it may be considered short‐term additive therapy for severe symptoms refractory to 1 or more antihistamines at maximum doses.^16^

## 4 CONCLUSION

While the most common COVID‐19 infection presentation is respiratory symptoms, physicians should not ignore other uncommon presentations like dermatological findings and cutaneous manifestation, which are increasingly being seen. Therefore, the management of urticaria is focused on controlling symptoms and typically includes parenteral and oral antihistamines and oral corticosteroids.

## AUTHOR CONTRIBUTIONS

UA, SU, and AJN involved in data collection, literature search, and manuscript preparation. All authors read and approved the final manuscript.

## CONFLICT OF INTEREST

The authors declare that they have no competing interests.

## ETHICS APPROVAL AND CONSENT TO PARTICIPATE

The article describes a case report. Therefore, no additional permission from our Ethics Committee was required.

## CONSENT

Written informed consent was obtained from the patient to publish this report in accordance with the journal's patient consent policy.

## Data Availability

All data generated or analyzed during this study are included in this published article.
